# Early Detection and Outcome in Borderline Personality Disorder

**DOI:** 10.3389/fpsyt.2019.00710

**Published:** 2019-10-09

**Authors:** Paola Bozzatello, Silvio Bellino, Marco Bosia, Paola Rocca

**Affiliations:** Department of Neuroscience, University of Turin, Turin, Italy

**Keywords:** borderline personality disorder, prodromal factors, early symptoms, childhood, adolescence, outcome

## Abstract

Borderline personality disorder (BPD) is a severe and heterogeneous mental disorder that is known to have the onset in young age, often in adolescence. For this reason, it is of fundamental importance to identify clinical conditions of childhood and adolescence that present a high risk to evolve in BPD. Investigations indicate that early borderline pathology (before 19 years) predict long-term deficits in functioning, and a higher percentage of these patients continue to present some BPD symptoms up to 20 years. There is a general accordance among investigators that good competence in both childhood and early adulthood is the main predictive factor of excellent recovery in BPD patients. Some authors suggest that specific childhood personality traits can to be considered precursors of adult BPD, as well as some clinical conditions: disruptive behaviours, disturbance in attention and emotional regulation, conduct disorders, substance use disorders, and attention-deficit-hyperactivity disorder. Unfortunately, diagnosis and treatment of BPD is usually delayed, also because some clinicians are reluctant to diagnose BPD in younger individuals. Instead, the early identification of BPD symptoms have important clinical implications in terms of precocious intervention programs, and guarantees that young people with personality disorders obtain appropriate treatments. This review is aimed to collect the current evidences on early risk and protective factors in young people that may predict BPD onset, course, and outcome.

## Introduction

Borderline personality disorder (BPD) is a severe and heterogeneous mental disturbance connoted by a pattern of identity diffusion, interpersonal disturbances, and chronic instability, with episodes of severe affective and impulsive dyscontrol (1). Personality disorders (PD) do not suddenly emerge in the adulthood; in fact, prodromal signs and processes that confer vulnerability to later personality pathology are already present in young age, often in adolescence (2–5). In adolescents, epidemiological data reported a point prevalence around 0.9%, but studies in this age group are still scarce (6). Cumulative prevalence rates of BPD in youths are respectively 1.4% and 3.2% at 16 years and at 22 years. In mental health setting, the diagnosis of BPD in adolescence reach a prevalence of 11% in psychiatric outpatients and up to 50% in inpatients (2, 6–8). Investigations indicate that early borderline pathology (before 19 years) predicts long-term deficits in functioning, and a higher percentage of these patients continue to present some BPD symptoms up to 20 years. (9) A considerable proportion of these individuals continue to suffer from borderline symptoms up to 20 years (10).

For this reason, clinical conditions of childhood and adolescence that present a high risk to evolve in BPD should be carefully monitored. Unfortunately, diagnosis and treatment of BPD is usually delayed as some symptoms are underestimated and clinician have hesitation to diagnose BPD in younger individuals. Stigma, the incompleteness of personality development in this age group, and similarities between physiological adolescent upheaval and BPD symptoms are the main reasons for this reluctance (11). Indeed, early identification of BPD symptoms may promote early intervention programs that should guarantee appropriate treatments in young people. Some retrospective studies in adult patients (12, 13) showed that the mean age of first psychiatric contact was 17 to 18 years and that the common failure in the diagnosis at first presentation resulted in losing the opportunity to set up early interventions. Several factors, including precocious environmental factors, child and adolescent temperamental characteristics, early psychopathological features, and neurobiological correlates were identified as predictors of early BPD onset. Although the importance of an early diagnosis to improve long-term outcome of the disorder is widely accepted, this issue is not extensively studied and many questions still remain open. In order to improve our knowledge on risk factors in young people that may predict early BPD onset, course, and outcome, we conducted a review to collect and summarize the available evidence in literature.

## Methods

In October 2018, an electronic search on PubMed about early prodromal factors and precursors of BPD without any filter or MESH restriction was performed, using the following search string: “borderline personality disorder” AND “early symptoms” OR “borderline personality disorder” AND “precursors” OR “borderline personality disorder” AND “prodromal factors” OR “borderline personality disorder” AND “childhood” OR “borderline personality disorder” AND “adolescence” OR “borderline personality disorder” AND “early symptoms” AND “outcome.” This string ensured a high sensitive search for the published works indexed in PubMed. A limitation of this review is that PubMed was the only database used to search the articles. Overlapping studies were excluded. We included the following types of publications: controlled trials, observational studies, longitudinal and prospective studies, cohort studies, and reviews from January 2000 until November 2018. Publications must concern early factors that predict BPD in young age as the main topic. We excluded publications written in a language other than English.

## Results

The search described in the previous section provided 2,193 records, and among them 1,788 overlapping studies were excluded. Total records included in the review were 405. Eligibility status for articles was determined in the following way: 1) all studies were screened on the basis of title and abstract; 2) papers that have passed the initial screening were reviewed on the basis of a careful examination of the full manuscript content. Three hundred and four were excluded because they did not fit the objective of the review, 19 because were not written in English, 3 for the lack of the complete manuscript. Thus, this review included 79 records, including 7 reviews, 51 longitudinal/prospective studies, 3 retrospective studies, 1 observational study, 1 commentary/expert article, and 16 controlled trials.

BPD symptoms and diagnosis were assessed with the following evaluation instrument: in the majority of cases for adult was used the official tool of DSM (Structured Clinical Interview for DSM-IV Axis II Personality Disorders SCID-II, and for DSM-5 Personality Disorders SCID-PD). Specifically for children and adolescents, most of the studies adopted the Borderline Personality Features Scale for Children (BPFS-C) (14) including a newly developed parent report version of the measure (BPFS-P) (15).

Number of studies participants ranged between 40 and 6,050. Seven studies included only females; one study included only males; 3 studies did not report the gender percentage; the remaining studies had an equal distribution of males and females. The vast majority of patients in the reviewed articles was Caucasian and this is a limitation both in terms of clinical and socio-cultural limitations. Duration of the longitudinal/prospective studies presented a wide range between 1 and 30 years. Ninety percent of studies enrolled participants from the community (40% of these were “high-risk” subjects on the basis of the presence of relevant risk factors, i.e. economic disadvantages), 10% from the clinical settings. Drop-out rates were acceptable, with a retention ranged between 43% and 96%. Majority of studies had a retention ≥70%.

**Figure 1:**
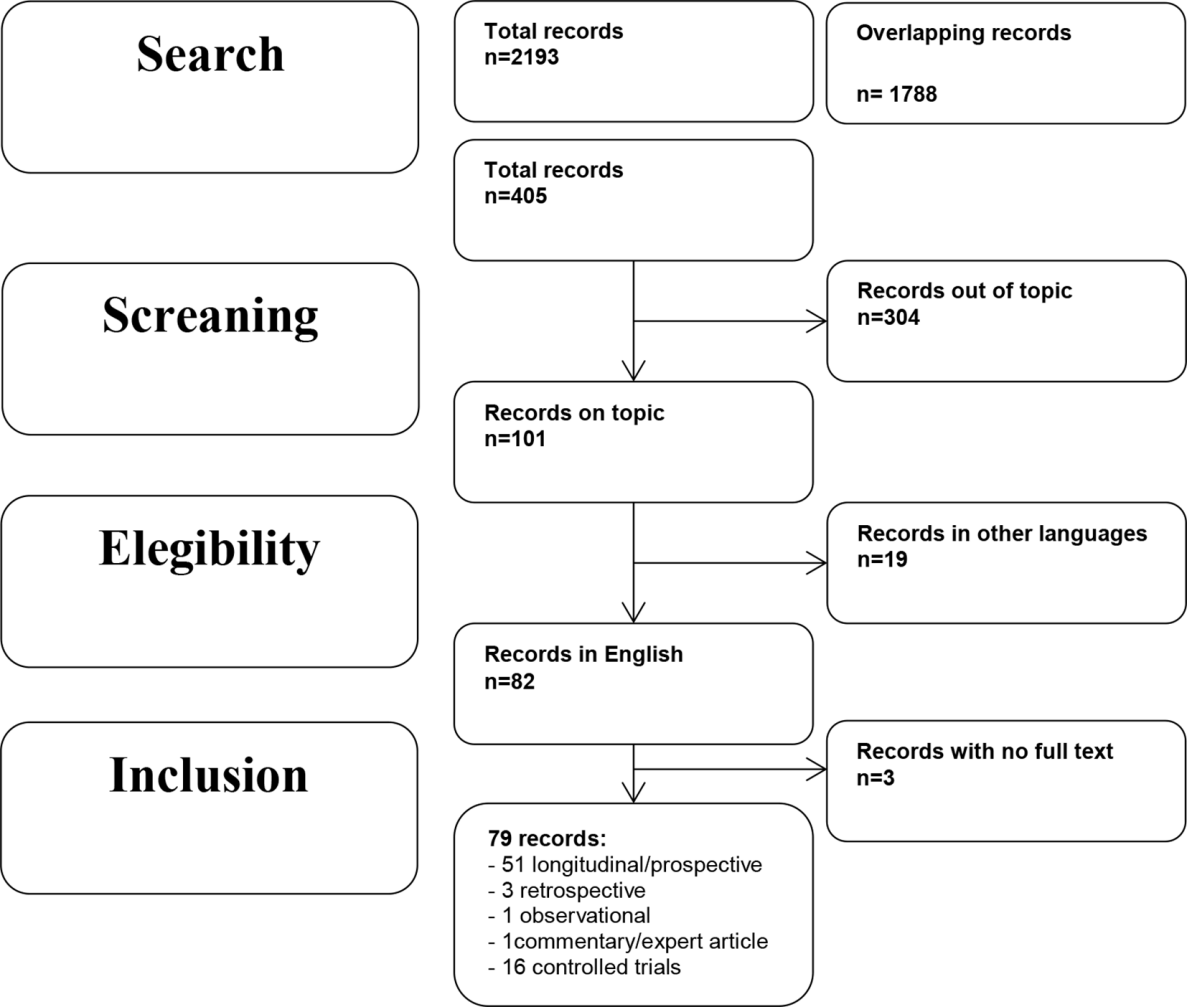
Literature search flowchart.

The selection process and a schematic representation of the results are represented in the literature search flowchart (**Figure 1**).

## Discussion

### Precocious Environmental Factors

Several studies have identified a broad range of environmental factors that are related to subsequent risk for BPD, including socio-economic status, family psychopathology, parent-child relationship, and maltreatments or other traumatic events. In recent years, a growing number of investigations has been focused on the powerful role of social influences, particularly bullying and rejection by peer groups.

#### Family-Related Factors and Early BPD

Only two longitudinal studies specifically investigated the association between socioeconomic status (low income, low educational level, and low status occupation) and early onset of BPD. In the study performed by Cohen and collaborators (16) the authors examined the effects of familial socioeconomic status on the severity of schizotypal and borderline personality disorders symptoms in a general population of 608 children and adolescents living in urban, suburban, and rural residence. These subjects were longitudinally studied between ages 10 and 36. The authors concluded that lower socioeconomic status predicted BPD symptoms and the effect of magnitude remained stable over time. The same results were found in the second study with a similar design and objective in a large community sample of 766 children (17).

Other investigations evaluated the impact of family environment including economic adversities and parents’ psychopathology on precocious onset of BPD. Four studies were aimed to verify the association with poverty and maladaptive behaviours, such as hitting, shouting, hostility, and parent conflicts, on early BPD. One study was conducted in a large sample of 6,050 mothers and their children recruited in the community (18), while three investigations were performed in samples (ranged between 113 to 2,282 participants) of high-risk subjects (19–21). Winsper and colleagues (18) observed mothers and children for 12 years and found that family adversities and maladaptive parental behaviours predicted increased risk for BPD in very young age (11 years). Stepp and colleagues (19–21) showed that poverty condition that required public assistance may predict BPD symptoms during adolescence.

Theories on the role of parents’ psychopathology, in particular maternal BPD, as putative precursor to BPD in children and adolescents (22, 23), have found empirical support from three longitudinal studies (24–26) and one controlled study (27). Barnow and colleagues (24) and Reinelt and collaborators (26) studied a large community sample (respectively, 286 and 295 subjects) during 5 years, while Stepp and colleagues’ study (25) included a sample of 816 subjects from the community who were observed for 16 years. Results were consistent in identifying maternal BPD as predictor of BPD onset in adolescence (15 years) (24, 26) and early adulthood (24 years) (25). Mahan and colleagues (27) evaluated the association between maternal BPD, maternal psychological control, and onset of BPD in adolescence. The authors sampled 28 mothers with a diagnosis of BPD, 28 control comparisons, and their adolescent offspring. All subjects were assessed for borderline features. Maternal psychological control was found positively associated with borderline features of mothers and with affective instability of offspring with an increased risk for adolescents of developing BPD themselves.

The impact of other maternal psychopathological dimensions on BPD onset in adolescents was evaluated. In a study conducted in a high-risk sample of 700 youths that were studied from mid-adolescence to young adulthood, authors observed that maternal externalizing disorder and offspring internalizing disorder were significant associated with BPD risk (28). Study performed by Winsper and colleagues (9) showed that maternal anxiety and depression during pregnancy predict early BPD in sons/daughters. In a similar way, depressive symptoms and antisocial personality disorder (ASPD) in caregivers predicted the onset of BPD in adolescence (14–17 years) in a sample of 2,212 high-risk subjects (20). Actually, this relation was significant in bivariate analyses, but not in final analyses of Stepp’s study.

Other three longitudinal studies aimed to evaluate the effects of maternal ego integration and impulsivity, medical problems, and interpersonal disturbances in producing early BPD symptoms in children/adolescents did not find any significant association (17, 29, 30).

As regards relationship between parents and children, studies obtained controversial findings. Among seven investigations, four reported a significant association between dysfunctional parent-child relationship and development of precocious BPD symptoms. Stepp and colleagues (25) evaluated in a 16-year follow-up study whether cohesion, discord, and support in relationships had an impact on BPD onset in 816 subjects from the community. The authors found that mother-child discord predicted BPD at 30 years. In accordance with the environment-genes interactions theory, Hammen and collaborators (31) observed a significant association between low relationship quality and BPD onset at 20 years in 385 subjects who had a particular genotype for the oxytocin receptor gene (AA/AG). Higher level of role confusion and disoriented behaviours in parent-young adult interaction seems to predict early borderline symptoms, in particular self-injuries and suicidality in late adolescence (32). Moreover, in a naturalistic study on the effects of inadequate parent-child boundaries, relationships centered on guilt induction, psychological control, and triangulation (children who mediated parental marital conflict) were found associated with children’s BPD features in 301 adolescents with severe behavioural and emotional disorders (33). Divergent findings were reported by two studies that did not show any significant association between parent-child relationship and onset of BPD in young age (30, 34).

#### Trauma-Related Factors and Early BPD

The role of early traumatic events and maltreatments in the onset, course, and pathogenesis of BPD was extensively examined by several studies. The World Health Organization categorized maltreatment into physical neglect, emotional neglect, emotional abuse, physical abuse, and sexual abuse (35). Children who are abused and/or neglected show deficits of functioning in several mental areas that are associated with BPD symptoms (36–40). Among 15 investigations on this topic, 5 did not report a significant correlation between maltreatment/trauma and BPD symptoms in childhood and adolescence. On the contrary, in 10 longitudinal studies with a duration ranged between 8 and 30 years in large community samples of children and adolescents (ranging between 113 and 2,764 participants) a significant relation was reported between early BPD onset and emotional and physical neglect and verbal abuse (30, 41–43); cumulative traumas (15); emotional abuse (44); physical abuse (15, 30, 45); sexual abuse (15, 20, 30, 44). Lyons-Ruth et al. (46) also stated that “abuse experiences could not account for the independent effect of early maternal withdrawal on borderline symptoms.” It is required that both abuse and these features of early caregiver–child interaction are present and produce their effects. Experiences of child abuse and neglect reciprocally interact with genes expressions influencing the emergence and timing of normal developmental processes and predicting child or adolescent borderline personality (47). Cicchetti and colleagues evaluated expression of the oxytocin receptor (OXTR) and the FK506 binding protein 5 (FKBP5) gene polymorphisms among 1,051 maltreated and non-maltreated children. Findings underlined the importance of the interaction between the genetic variants associated and maltreatment experiences in increasing the risk for early borderline symptomatology. Moreover, these associations were different between females and males (48). Females were more at risk for borderline symptoms when they add minor alleles of the two candidate genes. In contrast, males presented an increased for borderline symptoms when they presented major alleles. It is noticeable that the maltreatment-gene-gender interaction for females is consistent with a diathesis-stress model. In contrast, a different picture can be identified in males: frequent crossover interactions suggested a differential sensitivity to environment model.

In more recent years, particular attention was paid to the role of social group interactions, in particular peer relationships, in the development of psychiatric symptoms in childhood and adolescence. Dysfunctional relationships with peer may contribute to or promote the onset of BPD (2, 49). Being bullied during childhood predicted high risk to develop BPD not only in adulthood (2) but also in early adolescence (50–54). Five longitudinal studies specifically investigated this topic. Wolke and collaborators (50) participated in a 12-year prospective study that recruited 6,050 mothers and offspring enrolled in the Avon Longitudinal of Parents and Children (ALSPAC) study. Findings showed that chronic exposure to peer victimization during childhood can be considered a risk factor for the development of BPD symptoms in childhood (12 years). Among the same subjects of ALSPAC, Lereya and collaborators (51) evaluated the effect of exposure to bully between 7 and 10 years of age in 4,810 children and adolescents. Authors concluded that being bullied during childhood increased the risk of self-injuries in late adolescence, particularly if there is a concomitant exposure to an adverse family environment. ALSPAC data was also used by Winsper and colleagues (52) to assess the relationships between childhood disregulated behaviours, environmental factors (including bully victimization), and presence of BPD symptoms at 11 years. Bully victimization significantly predicted BPD, depressive and psychotic symptoms in children who had disregulated behaviours. Similar findings were obtained by data from 875 participants to the McMaster Teen Study (53), in which the association between early BPD development and chronic bullying involvement was confirmed in children with a reactive temperament. Antila et al. (54) verified the association of bullying behaviour in adolescence and PDs in early adulthood with particular attention to gender differences in 508 inpatient adolescents. They concluded that female, but not male, victims of bullying had a fourfold increased risk to develop PD, including BPD, in young age.

In summary, among precocious environmental factors, the most strong associations with the early onset of BPD are represented by verbal, physical, sexual abuses, maternal withdrawal/neglect in childhood, and chronic exposure to peer bully victimization during infancy. In addition, a smaller number of studies in a wide sample of patients monitored for many years showed that maternal psychopathology (BPD and depression), economic adversities, and maladaptive parental behaviours promoted the early development of BPD in the offspring. It remains open and understudied how genetic factors may interact with the environmental factors in promoting precocious BPD symptoms. Results are displayed in **Table 1**.

**Table 1:** Summary of studies on precocious environmental factors.

Family related	Study design	Patients (n)/ recruitment age	Trial duration	Outcomes
Cohen et al. (16)	Longitudinal study; Community population	680; 9–18 years	22 years	Lower SES predicted BPD symptoms and effect magnitude remained stable over time
Crawford et al. (17)	Longitudinal study; Community population	766; At birth	20 years	Lower SES predicted BPD symptoms
Winsper et al. (18)	Longitudinal study; Community population	6050; At birth	12 years	Family adversity predicted BPD symptoms
Stepp et al. (19)	Longitudinal study; Community high-risk	2282 girls; 14–19 years	14 years	Receipt of public assistance predicted BPD symptoms across adolescence
Stepp et al. (19,)	Longitudinal study; Community high-risk	2212 girls; 14–17 years	4 years	Receipt of public assistance predicted BPD symptoms; Caregiver ASPD and depression predicted BPD (*bivariate analyses only*)
Stepp et al. (21)	Longitudinal study; Community high-risk	113 girls; 5 years	10–13 years	Family adversity predicted increases in BPD symptoms
Barnow et al. (24)	Longitudinal study; Community population	286; 10 years	5 years	Maternal BPD predicted offspring BPD symptoms at 15 years old.
Reinelt et al. (26)	Longitudinal study; Community population	295; 10 years	5 years	Maternal BPD symptoms predicted offspring BPD symptoms at 15 years old and this association was mediated by maladaptive parenting style/behavior
Stepp et al. (25)	Longitudinal study; Community population	816; 14–18 years	16 years	Maternal BPD and paternal substance use predicted offspring BPD symptoms at 24. Mother-child discord predicted BPD symptoms
Mahan et al. (27)	Controlled trial; Community population	28 BPD mothers; 28 ctrl mothers; adolescent sons (14–18 years)		Maternal psychological control positively associated with all mothers’ BPD features and with adolescent affective instability with an increased risk for adolescents of developing BPD themselves
Conway et al. (28)	Longitudinal study; Community high-risk	700; 15 years	5 years	Maternal externalizing disorders and offspring internalizing disorders predicted BPD symptoms
Winsper et al. (99)	Longitudinal study; Community population	6050; At birth	12 years	Maternal anxiety and depression during pregnancy predicted BPD symptoms
Hammen et al. (31)	Longitudinal study; Community High Risk	385; 15 years	5 years	Relationship quality & oxytocin receptor genotype interacted to predict BPD symptoms: relationship quality predicted BPD symptoms for those with AA/AG genotype, not GG genotype
Lyons-Ruth et al. (32)	Longitudinal study; Community population	120; 20 years	21 years	Role confusion and disoriented behaviours in parent-young adult interaction predicted early BPD symptoms
Vanwoerden et al. (33)	Naturalistic study; community inpatients	301; 12–17 years		Relationships centered on guilt induction, psychological control and triangulation predicted BPD symptoms
Trauma-related factors	Study design	Patients (n)/ recruitment age	Trial duration	Outcomes
Johnson et al. (41)	Longitudinal study; Community population	738; <18 years	17 years	Supervision neglect predicted BPD symptoms
Johnson et al. (42)	Longitudinal study; Community population	793; 5 years	17 years	Verbal abuse predicted BPD symptoms
Carlson et al. (30)	Longitudinal study; Community high-risk	162;	28 years	Physical and sexual abuse predicted BPD symptoms
Jovev et al. (43)	Longitudinal study; Community population	245; 11–13 years	2 years	Abuse associated with BPD symptoms for children with low Affiliation
Cohen et al. (16)	Longitudinal study; Community population	680; 9–18 years	22 years	Cumulative trauma (physical and sexual abuse & other traumas) predicted BPD symptoms.
Bornovalova et al. (44)	Longitudinal study; Community population	2764;	7–13 years	Abuse (physical, sexual, emotional) predicted BPD symptoms
Belsky et al. (45)	Longitudinal study; Community population	1116; 5 years	7 years	Physical abuse predicted BPD symptoms
Stepp et al. (21)	Longitudinal study; Community high-risk	113; 16 years	3 years	Sexual abuse predicted BPD symptoms
Lyons-Ruth et al. (46)	Longitudinal study; Community high-risk	56; At birth	21 years	Concomitance of childhood abuse and maternal withdrawal predicted BPD symptoms
Wolke et al. (50)	Longitudinal study; Community population (ALSPAC)	6050; at birth	12 years	Chronic exposure to peer victimization predicted BPD symptoms
Lereya et al. (51)	Longitudinal study; Community population (ALSPAC)	4810; at birth	18 years	Bullying exposure increased risk of self-harm by exacerbating the effects of exposure to an adverse family environment
Winsper et al. (52)	Longitudinal study; Community population (ALSPAC)	4826; at birth	14 years	Bully victimisation predicted BPD, depression, and psychotic symptoms
Haltigan and Vaillancourt (53)	Longitudinal study; Community population (McMaster Teen Study)	875; 10 years	6 years	Association between early BPD development and chronic bullying involvement in children with a reactive temperament
Antila et al. (54)	Longitudinal study; Clinical inpatients	508; 13–17 years	12 years	Increased (fourfold) risk for bullied female to develop PD, mostly BPD

ALSPAC, Avon Longitudinal Study of Parents and Children; ASPD, antisocial personality disorder; BPD, borderline personality disorder; ctrl, control; PD, personality disorder; SES, socioeconomic status.

### Child and Adolescent Temperament and Personality Factors

The investigation of intrapsychic factors, including temperamental characteristics and personality trait profiles in childhood and adolescence, is fundamental to recognize predictors of BPD at an early phase. Researchers identified several personality traits in children or adolescents, including affective instability, negative affectivity, negative emotionality, inappropriate anger, poor emotional control, impulsivity, and aggression, that could prepare to borderline pathology [e.g., Refs. (45, 55–57)]. Few studies evaluated the relation of childhood personality traits to BPD in adulthood (30, 44, 58). Fifteen investigations examined the relationship between temperament or personality features associated with early BPD symptoms. Only one study (30) did not find any significant association in the final analyses.

Two studies adopted the Cloninger’s model to evaluate the association between temperamental patterns in childhood or adolescence and onset of BPD (59, 60). In the first study (59) temperamental characteristics were retrospectively collected in 180 depressed adult patients with personality disorders. Although it is hard to distinguish temperamental dimensions in personality of adult patients, authors found that high harm avoidance and novelty seeking (in combination with childhood experiences and adolescent psychopathology) can be considered predictive of early BPD. In line with this investigation, Kaess et al. (60) observed in a controlled study comparing 33 BPD adolescents, 35 clinical controls, and 31 healthy subjects that high harm avoidance and novelty seeking but low reward dependence represent a biological vulnerability for developing BPD.

Across other temperamental traits, aggressive behaviors in childhood and early adolescence was associated to onset of BPD. Crick and collaborators (55) investigated different subtypes of aggression in a prospective study that recruited 400 children and found that relational aggression, but not physical aggression, emerged as a significant predictor for BPD features. This result was confirmed by Underwood (61) in a prospective study with the same objective. Similarly, Cramer et al. (62) performed a longitudinal study, in which childhood personality traits were assessed at age 11 in 100 subjects and provided evidence that aggression and impulsivity are two predictive traits for BPD traits at 23 years. Vaillancourt and colleagues (57) prospectively found in 484 children and adolescents that aggression predicted the diagnosis of BPD at 14 years with some gender differences: relational aggression was the predominant predictor in boys, while physical aggression was the strongest predictor in girls.

Negative emotionality, in terms of negative affectivity and poor emotional control, is another important precocious factor associated to BPD onset. Lenzenweger and collaborators (22) conducted a community 3-year study with 250 adolescents/young adults, aimed to evaluate whether negative emotionality and other dimensions such as affiliation, constraint, and agency might impact on early onset of BPD. Findings showed that negative emotionality and low constraint predicted BPD at 19 years, and lower agency predicted increasing of BPD during time. Tragesser and collaborators (63) in a high-risk population of 353 subjects of 18 years reported a significant association of negative affectivity and impulsivity in childhood with BPD at 20 years. Similar findings were obtained by Stepp and collaborators in two following investigations (19, 20) with a larger sample ranged between 2,212 and 2,282 children/adolescents. They confirmed the role of negative affectivity and impulsivity in predicting BPD, even at 14 years (19), and highlighted the importance of higher activity and lower sociability in childhood as precursors of the disorder. As concerns negative emotionality, the result was replicated in two following studies (5, 64) with similar design and number of participants. In addition, Hallquist and colleagues (64) found that low self-control may predict BPD at 14 years and a worsening self-control increased BPD symptoms during the time.

Low self-control, impulsivity, and affective instability are three tightly connected dimensions that in very young age can be considered predictors for developing borderline pathology. Several investigations have assessed the influence of these constructs in childhood on later BPD symptoms. Tragesser and colleagues (65) reported that affective instability and impulsivity predicted BPD onset at 20 years. Gratz et al. (66) highlighted, in a sample of 263 children (9–13 years), the importance of interrelationship among these two relevant personality traits (affective instability and impulsivity) with low self- and emotion regulation, and with childhood borderline personality symptoms. Lower self-control and higher level of impulsivity were also identified as predictors of a diagnosis of BPD at 12 years in a 7 years twins study conducted in 1,116 children (around 5 years old) (45).

Only one study investigated the impact of anger, as temperamental trait, in childhood on BPD in adolescence/adulthood. Crawford and colleagues (17) showed a significant association between anger/tantrum dimension and BPD symptoms in 766 children who were followed for 20 years.

Five studies explored the interaction between child/adolescent personality traits and environmental or neurobiological factors in development of precocious BPD. Four investigations examined the effect of the relationships between temperamental characteristics and childhood maltreatment on the onset of BPD. Jovev et al. (43) studied the interaction between emotional control and affiliation traits, parental maltreatment and BPD in 245 children aged between 11 and 13 years. They observed that specific early temperamental features, particularly low emotional control, interact with familial maltreatment in promoting BPD symptoms across early to middle adolescence. On the other hand, parental abuse could have a moderating role in the presence of low affiliation. Martin-Blanco and colleagues (67) found in 130 subjects with early BPD that neuroticism-anxiety, aggression-hostility dimensions, and emotional abuse were independent risk factors associated with BPD. Two studies of the same year performed by Sharp et al. (68) and Stepp et al. (21) with different sample amplitude and duration, respectively, followed 730 adolescents for 1 year and 113 adolescents studied for 13 years and reported that the effect of lower self-control in promoting early onset of BPD was mediated by harsh familial discipline (68), and the impact of negative affectivity on early BPD was moderated by family adversities (21).

One study evaluated in 153 healthy adolescents the interaction of a temperamental risk factor and a neurobiological risk factor in predicting the emergence of BPD during early adolescence (69). Authors examined several temperamental factors and volumetric measures of hippocampal asymmetry. Results showed that subjects were more likely to have BPD symptoms in presence of high affiliation, low effortful control, and rightward hippocampal asymmetry.

In summary, temperamental traits in childhood, including relational aggression, impulsivity, low emotional control, and negative affectivity, are robust predictors of early onset of BPD. Some evidences support the role of the interaction between temperamental features (low emotional control, negative affectivity, and low affiliation) and familial environment (parental maltreatment, harsh discipline, and familial adversities) in developing BPD.

### Early Psychopathological Features and Diagnosis

Available evidences highlighted that internalizing and externalizing psychopathology is often present before the onset of BPD in adolescents. Externalizing pathology includes conduct disturbances, oppositional defiant disorder, attention-deficit/hyperactivity symptoms, impulsive-aggressive behaviours, self-injuries, and substance use disorder; while internalizing pathology mainly involves depression and anxiety, but also dissociation and suicidality. In addition, obsessive–compulsive disorder, separation anxiety disorder, and social phobia were frequently observed in adolescent populations (2, 11, 70, 71). Some authors suggested that internalizing and externalizing disorders emerge in pre-adolescence as anxiety and depressive symptoms in females, and ADHD, conduct problems in males. These disorders may form a platform on which develops personality pathology during adolescence (72, 73). In the context of predisposing biological vulnerabilities and interacting stressful life events, these antecedent disorders represent a predisposing condition that, if untreated, may contribute to the onset of personality pathology during adolescence (73).

Seventeen investigations explored the psychopathological conditions predicting BPD in youths. Three of them did not find any significant association. One study investigated the effect of interaction of negative emotionality and internalizing psychopathology on early onset of BPD (25). Conway and collaborators (28) combined risk factors into a more comprehensive developmental model of borderline pathology in a community sample of 815 youths (15 years of age) at high risk for psychopathology due to maternal depression. In fact, they examined the effects between several environmental stressors, including occurrence of acute stressors and chronic stressors across individual, family, peer, and academic contexts, and personal characteristics to give a contribution to the hypothesis that BPD results from the complex interaction between pathogenic environments and individual vulnerabilities. Results showed that only adolescent internalizing psychopathology and trait of negative affectivity continued to predict borderline pathology after controlling for the presence of other risk factors. Krabbendam and colleagues (74) identified dissociation (internalizing symptom) significantly associated with onset of BPD at 20 years in a prospective study in which 184 adolescents were followed for 6 years. Self-injuries, another symptom related to internalizing psychopathology, was found predictive of early BPD in one investigation performed in 77 adolescent psychiatric inpatients and 50 young detainees (75). Sharp and colleagues (68) in a 1-year study including 730 adolescents (16 years) found that anxiety and depression (internalizing symptoms) predicted BPD at 17 years. Depression recurred as predictor of early BPD in other three studies (25, 76, 77) in samples including respectively 158, 524, and 816 subjects aged between 14 and 17 years. Studies lasted from 8 to 16 years of follow-up. In these investigations were identified as predictors of early BPD substance use disorder (25, 76, 78) and attention deficit hyperactivity disorder (ADHD) (77). It is noticeable that both internalizing and externalizing disorders are implicated in promoting BPD in young patients. Belsky and collaborators (45), Bornovalova and colleagues (78), and Bo and Kongerslev (79) confirmed the role of both internalizing and externalizing psychopathological conditions to predict early BPD. In particular, Bo and Kongerslev (79) compared 46 children and adolescents with BPD and 62 children and adolescents with other clinical conditions. Findings showed that high level of psychopathology (internalizing and externalizing), poor mentalizing abilities, and attachment problems were strictly associated to BPD in adolescents compared with psychiatric disorders other than BPD. In addition, Bornalova et al. (78) reported that higher number of BPD traits predicted earlier onset and faster worsening of substance use symptoms and that substance use slows the reduction of BPD traits in youths.

Some studies showed a significant association between externalizing pathologies and early onset of BPD. Miller and colleagues (80) observed a significant relationship between ADHD in childhood and BPD at 18 years in a 10 years follow-up study including 181 children. Two following studies (71, 81) confirmed this association and also identified the oppositional defiant disorder in childhood as predictor of BPD respectively at 24 and 14 years. Similar findings were observed by Stepp and colleagues (20) that found a significant relationship of adolescent opposite defiant disorder and conduct disorder with BPD onset at age ranged between 14 and 17 years.

In the study performed by Wolke et al. (50) and described in the previous section, it was found that any Axis I diagnosis predicted BPD at very young age of 12 years. A recent controlled study performed by Thompson et al. (82) evaluated the prevalence of psychotic-like symptoms in 171 subjects of 15–18 years with BPD features. The authors found that adolescents with full-threshold BPD presented more confusion, paranoid ideation, visual hallucinations, and odd thoughts than adolescents with sub-threshold BPD symptoms and adolescents with no BPD symptoms.

In summary, among early psychopathological factors, both internalizing and externalizing disorders in childhood and adolescence are involved in producing early BPD in adult. In particular, the most robust associations are represented by depression, substance use disorder, ADHD, and oppositional defiant disorder. As the precise role of each of these potential etiological factors in determining risk for BPD is still unclear and there is a degree of overlap between them, their interaction with environmental stress has to be carefully considered. An additional hypothesis to explain the overlap of internalizing and externalizing disorders is that BPD pathology expresses itself in early stages of the disorder mainly with externalizing behaviours, although features of internalizing disorders are also present. When BPD adolescents grow up behavioral manifestations of externalizing disorders diminish in favour of a stronger expression of internalizing pathology (83). Result are displayed in **Table 2**.

**Table 2:** Summary of studies on child and adolescent temperament and personality factors and early psychopathological features.

Temperament and personality factors	Study design	Patients (n)/ recruitment age	Trial duration	Outcomes
Joyce et al. (59)	Retrospective study; Clinical outpatients	180 depressed		High NS and HA (in combination with childhood experiences and adolescent psychopathology) predictive of early BPD
Kaess et al. (60)	Controlled trial; Clinical patients and community population	33 BPD, 35 CC, 15 31 HC; 13-19 years		High NS and HA and low RD biological vulnerability for developing BPD
Crick et al. (55)	Longitudinal study; Community population	400	1 year	Relational aggression predicted BPD symptoms
Underwood et al. (61)	Longitudinal study; Community population	255; 9 years	5 years	High social aggression in female predicted BPD symptoms
Cramer et al. (62)	Longitudinal study; Community population	100; 11 years	12 years	Impulsivity and aggression predicted BPD symptoms
Vaillancourt et al. (57)	Longitudinal study; Community population	484; 10 years	4 years	Aggression (relational in boys, physical in girls) predicted BPD symptoms
Lenzenweger et al. (22)	Longitudinal study; Community population	250;	3 years	Negative emotionality and low constraint predicted BPD at 19 years, and lower agency predicted increasing of BPD
Tragesser et al. (63)	Longitudinal study; Community high risk	353 years; 18 years	2 years	Negative affectivity and impulsivity predicted BPD symptoms
Stepp et al. (19)	Longitudinal study; Community high-risk	2282 girls; 14–19 years	14 years	Higher activity and lower sociability predicted increases in BPD symptoms, higher shyness predicted decreases in BPD symptoms
Stepp et al. (20)	Longitudinal study; Community high-risk	2212 girls; 14–17 years	4 years	Negative affectivity and impulsivity predicted BPD symptoms
Hallquist et al. (64)	Longitudinal study; Community high-risk	2228 girls; 5–8 years	10 years	Poor self-control predicted BPD symptoms at 14 ys and a worsening self-control increased BPD symptoms during time
Tragesser et al. (65)	Longitudinal study; Community high-risk	350; 18 years	2 years	Affective instability and impulsivity predicted BPD symptoms at 20 ys
Gratz et al. (66)	Retrospective study; Community population	263; 9–13 years		Significant interrelationship among affective instability and disinhibition, self- and emotion regulation deficits, and childhood borderline personality symptoms
Belsky et al. (45)	Longitudinal study; Community population	1116; 5 years	7 years	Lower self-control and higher impulsivity predicted BPD dx at 12 ys
Crawford et al. (17)	Longitudinal study; Community population	766; At birth	20 years	Anger/tantrums predicted BPD symptoms
Jovev et al. (43)	Longitudinal study; Community population	245; 11–13 years	3 years	Low emotional control robust predictor in developing BPD symptoms; parental abuse moderating role in the presence of low affiliation
Martin-Blanco et al. (67)	Retrospective study; Clinical inpatients	130		Neuroticism-anxiety and aggression-hostility dimensions, as well as emotional abuse, independently associated with BPD
Sharp et al. (68)	Longitudinal study; Community population	730; 16 years	1 year	Lower self-control predicted BPD symptoms via harsh familial discipline
Stepp et al. 2015	Longitudinal study; Community high-risk	113 girls; 5 years	10–13 years	Higher levels of negative affectivity and family adversity predicted BPD symptoms
Jovev et al. (69)	Controlled trial; Community high-risk	153; 11–13 years		BPD symptoms associated to high affiliation, low effortful control and rightward hippocampal asymmetry (differences between genders)
Early psychopathological features	Study design	Patients (n)/ recruitment age	Trial duration	Outcomes
Conway et al. (28)	Longitudinal study; Community high-risk	700; 15 years	5 years	Adolescent internalizing psychopathology and trait of negative affectivity predicted BPD symptoms
Krabbendam et al. (74)	Longitudinal study; Clinical incarcerated	184 girls; 16 years	3–6 years	Dissociation predicted BPD diagnosis at 20 ys
Koenig et al. (75)	Controlled trial; Clinical inpatients and incarcerated	77 inpatients; 16,6 mean age 50 detainees; 17,7		Self-injuries predicted BPD symptoms
Sharp et al., (68)	Longitudinal study; Community population	730; 16 years	1 year	Anxiety and depression predicted BPD symptoms at 17 ys
Ramklint et al. (76)	Longitudinal study; Clinical inpatiens	158; 15 ys mean age	16 years	MDD and substance use disorder predicted adult BPD diagnosis
Thatcher et al. (77)	Longitudinal study; Community population and clinical outpatients	355 CC; 169 HC; 16 ys mean age	8–12 years	MDD and ADHD predicted ‘severe’ BPD symptoms
Stepp et al. (25)	Longitudinal study; Community population	816; 14–18 years	16 years	Depression, substance use and suicidality predicted BPD symptoms
Belsky et al. (45)	Longitudinal study; Community population	1116; 5 years	7 years	Internalizing and externalizing conditions predicted early BPD
Bornovalova et al. (78)	Longitudinal study; Community population	1763 twins; 11–17 years	10 years	Higher levels of BPD traits contribute to earlier onset of substance use. Substance use slows the normative decline of BPD traits in youths
Bo and Kongerslev (79)	Controlled trial; Clinical outpatients	46 BPD; 62 CC; 13–18 years		High level of psychopathology, poor mentalizing abilities, and attachment problems were strictly associated to BPD compared to adolescents with psychiatric disorders other than BPD
Miller et al. (80)	Longitudinal study; Clinical outpatients	96 ADHD; 85 CC; 7–11 years	10 years	Childhood ADHD predicted BPD at 18 ys
Burke et al. (107)	Longitudinal study; Clinical outpatients	142 boys; 7–22 years	12–18 years	Oppositional-defiant disorder and ADHD symptoms through adolescence predicted BPD symptoms at 24 ys
Stepp et al. (81)	Longitudinal study; Community high-risk	1233 girls; 5–13 years	6–9 years	Oppositional-defiant disorder and ADHD symptoms predicted BPD symptoms at 14 ys
Stepp et al. (20)	Longitudinal study; Community high-risk	2212 girls; 14–17 years	4 years	Conduct disorder and oppositional-defiant disorder symptoms predicted BPD symptoms
Wolke et al. (50)	Longitudinal study; Community population (ALSPAC)	6050; at birth	12 years	Any Axis I diagnosis predicted BPD at 12 ys
Thompson et al. (82)	Controlled trial; Clinical outpatients	171; 15–18 years		Adolescents with full-threshold BPD reported more confusion, paranoia, visual hallucinations , and strange thoughts than the other two subgroups

ADHD, attention deficit hyperactivity disorder; ALSPAC, Avon Longitudinal Study of Parents and Children; BPD, borderline personality disorder; CC, clinical controls; dx, diagnosis; fts, features; HA, harm avoidance; HC, healthy controls; MDD, major depressive disorder; NS, novelty seeking; RD, reward dependence; ys, years.

### Neuroimaging and Early BPD

To date, no functional brain imaging studies have been published in adolescent populations with BPD. Neuroimaging studies of these subjects only focused on structural abnormalities, including both changes in grey and white matter.

It is interesting to evaluate the neurobiological underpinnings of younger populations with BPD symptoms at their beginnings in order to minimize the burden of confounders: some factors, such as prolonged duration of illness, pharmacotherapy, and recurring traumas, could themselves produce changes of brain structures (84, 85).

Orbitofrontal cortex (OFC) was found reduced in volume by two studies which compared BPD to control groups (84, 86). By means of region of interest (ROI) methodology, Chanen et al. (84) found that 20 BPD patients of 15–19 years showed a right-sided loss of OFC grey matter, reversing the normal (right > left) asymmetry of brain area volume, in comparison to 20 control subjects. In the study performed by Brunner et al. (86) using voxel-based morphometry (VBM) techniques, 20 BPD patients of 14–18 years displayed a significant shrinking of the left OFC and bilateral dorsolateral prefrontal cortex (DLPFC) compared with a group of 20 healthy controls. Authors found no differences between BPD group and 20 patients with other mental disorders. Using the same cohort of patients but varying imaging technics (diffusion tensor imaging, DTI), Maier-Hein et al. (87) found that the bilateral fornices of BPD group had lower myelination and their white matter bundles were less organized when compared to clinical and healthy controls. Thalamus and hippocampus, as well as the heteromodal association cortex, showed white matter disrupted connections in BPD patients. Such findings led the authors to argue that adolescents with BPD lack a normally functioning network involved in emotion processing. Reanalyzing the same data by means of another software, Richter et al. (85) found that BPD patients’ right amygdala was smaller than healthy (but not clinical) controls’ right amygdala. In the same study the authors demonstrated that hippocampal volume of BPD patients was the smaller in comparison to both control groups. In the same sample, Walterfang et al. (88) showed that BPD patients had the same dimension of corpus callosum as healthy controls.

Two studies reported a volume reduction of anterior cingulate cortex (ACC) (89, 90) in adolescents with BPD. In the study performed by Whittle et al. (89) a shrinking in left AC cortex volume (across limbic and paralimbic regions) was found in 15 female patients (mean age 17,39) with BPD compared to 15 controls (mean age 19,65). Goodman et al. (90) found that 13 BPD/major depressive disorder (MDD) patients (mean age 15,8) had smaller relative volume in a part of the ACC, Brodmann area 24, in comparison to healthy subjects (mean age 16,2).

A study performed by Jovev et al. (43) has already been cited in a previous paragraph (see child and adolescent temperament and personality factors). The most important finding of the study is the moderator role of atypical rightward hippocampal asymmetry in the relationship between temperament traits and BPD symptoms in adolescents aged between 11 and 13 years. High scores in both affiliation and atypical rightward hippocampal asymmetry were good predictors of BPD symptoms in boys. For girls, low effortful control was linked to strong BPD symptoms in the presence of atypical rightward hippocampal asymmetry. It is noticeable that abnormalities of hippocampus are involved in memory processes and in emotional response to memories (emotional regulation and emotional recognition).

In a Diffusion Tensor Imaging (DTI) study, New et al. (91) observed bilateral tract specific decreased fractional anisotropy (FA) in the inferior longitudinal fasciculus (fibre bundle connecting the temporal lobe and occipital lobe) in 14 BPD adolescents in comparison to 13 controls. Moreover, a lower FA in the uncinate and occipitofrontal fasciculi (the white matter tracts connecting parts of the limbic system to the OFC among other frontal regions) was found at follow-up analysis in BPD adolescents.

Mainly in accordance with adult findings, studies discussed above showed structural anomalies both in grey and white matter of frontolimbic areas that are deeply involved in emotion regulation and impulse control. Even if no functional studies on BPD adolescents have been carried out yet, white matter alterations are compatible with functional findings in adults (92) displaying disruption in frontolimbic system connectivity. Result are displayed in **Table 3**.

**Table 3:** Summary of studies on neuroimaging and effect of early detection on course and outcome of BPD.

Neuroimaging	Study design	Patients (n)/ recruitment age	Trial duration	Outcomes
Early detection effects	Study design	Patients (n)/ recruitment age	Trial duration	Outcomes
Chanen et al. (84)	Controlled trial; Clinical outpatients and community population	20 BPD; 20 HC; 15–19 years		Reversal of the normal (right > left) asymmetry of OFC grey matter volume in BPD pts compared with HC
Richter et al. (85)	Controlled trial; Clinical outpatients and community population	20 BPD pts; 20 CC; 20 HC; 14–18 years		Right amygdala, right and left hippocampi smaller in BPD pts compared to healthy (but not clinical) controls
Brunner et al. (86)	Controlled trial; Clinical outpatients and community population	20 BPD pts; 20 CC; 20 HC; 14–18 years		Left OFC and bilateral DLPFC smaller in BPD pts compared with HC, but not CC
Maier-Hein et al. (87)	Controlled trial; Clinical outpatients and community population	20 BPD pts; 20 CC; 20 HC; 14–18 years		Lower fractional anisotropy in the bilateral fornices of BPD group compared to CC and HC
Walterfang et al. (88)	Controlled trial; Clinical outpatients and community population	20 BPD; 20 HC; 15–19 years		No differences in corpus callosum size between BPD group and HCs
Whittle et al. (89)	Controlled trial; Clinical outpatients and community population	15 BPD girls; 15 HC girls; 15–19 years		Left ACC volume smaller in BPD pts compared to HC
Goodman et al. (90)	Controlled trial; Clinical outpatients and community population	13 BPD; 13 HC; 15,8 ys mean age		BPD/MDD patients had smaller BA 24 volume. Smaller BA 24 volume was associated with BPD (but not depressive) symptoms
Jovev et al. (69)	Controlled trial; Community high-risk and community population	153 11–13 years		BPD symptoms associated to high affiliation, low effortful control and rightward hippocampal asymmetry (differences between genders)
New et al. (91)	Controlled trial; Clinical outpatients and community population	14 BPD pts; 13 HC 15,8 ys mean age		Lower fractional anisotropy in the inferior longitudinal fasciculus, uncinate, and occipitofrontal fasciculi
Gunderson et al. (96)	Longitudinal study; Clinical outpatients	160 BPD pts; 18–45 years	2 years	Early history of abuse and neglect is associated with a poor prognosis
Winograd et al. (100)	Longitudinal study; Community population	748; 9–18 years	20 years	BPD in childhood and adolescence predictive of enduring impairment in interpersonal, occupational, and financial domains of functioning
Crawford et al. (101)	Longitudinal study; Community population	629; 13,8 ys mean age	20 years	Persistent poor functional outcome in BPD features adolescents, including increased risk for substance use and mood disorders, interpersonal dysfunctions, and poor quality of life
Biskin et al. (102)	Longitudinal study	49 girls; 19,6 ys mean age	4 years	Non-remitters BPD pts more likely to be unemployed and to have a current episode of major depressive disorder, lifetime substance use disorder, self-reported childhood sexual abuse, and being unemployed
Haltigan and Vaillancourt (53)	Longitudinal study; Community population	875; 10 years	4 years	Child-reported ADHD and somatization symptoms predicted elevated or rising trajectory, whereas parent-reported anxiety symptoms predicted intermediate or stable trajectory
Zanarini et al. (98)	Longitudinal controlled study; Community population	290 BPD pts; 72 Axis II pts	20 years	Axis II pts reached higher rates of both good and excellent recovery than BPD pts. Competence in both childhood and adulthood was the best predictor of attaining an excellent recovery

ACC, anterior cingulate cortex; ADHD attention deficit hyperactivity disorder; BA, Brodmann area; BPD, borderline personality disorder; CC, clinical controls; DLPFC, dorsolateral prefrontal cortex; HC healthy controls; OFC, orbitofrontal cortex; MDD, major depressive disorder; pts, patients; ys, years.

### Effect of Early Detection on Course and Outcome of BPD

Detecting personality abnormalities in childhood and adolescence is a challenge for clinicians and is crucial to increase our knowledge of personality psychopathology in adulthood. Several investigations suggested that generally BPD symptoms have their onset in adolescence, reach a peak in early adulthood, and then decline during the course of life (83, 93). The decrease of BPD symptoms might be attributed to declining levels of impulsivity and dyscontrolled behaviors, while the persistence of a subsyndromal BPD is probably due to enduring negative affects (94). Other studies indicated that 20% of youths had an increase of PD symptoms over the decade from mid-adolescence to early adulthood (95). Only a few studies specifically investigated the effect of early onset on outcome and whether early factors may influence the trajectories of later BPD. In a 2-years follow-up study Gunderson et al. (96) found that an early history of abuse and neglect is associated with a poor prognosis in 160 adults with BPD. Among factors related to a poor long-term outcome, younger age at first treatment plays an important role together with affective instability, length of prior hospitalization, antisocial behaviors, comorbid substance use disorder, history of family psychiatric diseases, and dysfunctional relationship with parents (97, 98). Available studies indicated that long-term (until 20 years) functioning does not reach a satisfactory level, even when BPD achieve the clinical remission (99). In particular, BPD in childhood and adolescence predicted a long-lasting impairment in relational, occupational, and economic domains, as resulted by investigation performed by Winograd and collaborators (100) in 748 subjects prospectively followed for 20 years. These findings are consistent with those obtained in the investigation published by Crawford and colleagues (101). The authors highlighted that poor functional outcome persists for many years in adolescents who presented borderline features, including risk for substance use, depressive symptoms, interpersonal dysfunctions, and poor quality of life. Furthermore, Biskin and colleagues (102) in a 4-years prospective study found that woman who received a diagnosis of BPD in adolescence (49 patients) were less likely to have a stable occupation in comparison with other psychiatric disorders. Haltigan and Vaillancourt (53) evaluated the associations of childhood risk factors and trajectories during 4 years of later BPD features in a 875 community-based sample. Authors identified three distinct trajectories on the basis of symptoms and severity of course of BPD: low or stable, intermediate or stable, and elevated or rising. Attention-deficit hyperactivity disorder (ADHD) and somatization symptoms reported by child predicted elevated or rising trajectory, whereas anxiety reported by parent and ADHD symptoms reported by child predicted intermediate or stable trajectory. Presence of somatization symptoms reported by child was the only factor to differentiate the intermediate or stable and elevated or rising trajectory groups and may predict histrionic traits and hypochondria in later BPD. Moreover, young subjects with a reactive temperament who experienced chronic bullying by peers were more likely to be in a rising/elevated BPD features trajectory group. In a recent long-term follow-up study, Zanarini and colleagues (98) examined two levels of positive outcome at 20 years: “good and excellent recovery” achieved by BPD patients in comparison with other personality disorders (controls). Results showed that controls reached superior rates of both “good and excellent recovery” than BPD patients and that high competence in both childhood and adulthood was the main predictor of excellent recovery. Predictors associated with competence were higher IQ, good childhood work competence, and temperamental features including neuroticism and agreeableness. In particular, pattern of lower neuroticism and higher agreeableness can be interpreted as protective temperamental factors in childhood that allow them to develop a stable and cohesive personality (98, 103, 104).

## Conclusions

On the basis of the results discussed in the previous paragraphs, adolescence represents a sensitive and vulnerable phase for the development of BPD (83). In order to identify and monitor high-risk population from premorbid manifestations it is important to characterize and detect main associated risk factors for early BPD (99, 105). Despite strong evidence supporting the benefits of early identification of BPD and the recommendations of treatment guidelines for BPD (10, 106), fear of stigmatization still constitutes a barrier to early diagnosis in clinical practice (2, 8). Different processes may contribute to the early onset of this personality disorder and several precocious risk factors are involved. Among family-related environmental factors, low socioeconomic status of family, economic adversities, and maladaptive behaviors in parents are three robust independent prospective risk factors for early BPD (16–21). Another significant precursor to BPD in childhood and adolescence is maternal psychopathology. The most significant result concerns the association between maternal BPD and offspring early BPD (24–26). The association between other maternal psychopathological conditions such as externalizing disorder history (28) and anxiety (9) with early BPD onset is still understudied. As concerns the relationships between parents and children, investigations obtained controversial results. Anyway, some kind of dysfunctional parent-child relationship was identified as a potential predictor of early BPD: discord between mother and child, significant role confusion, and disoriented behaviors in parents, inadequate parent-child boundaries, psychological control by parents, and low relationships quality in individuals with a particular genotype for the oxytocin receptor gene (25, 31–34). Among trauma-related environmental factors, verbal abuse, emotional abuse, physical abuse, sexual abuse, and emotional and physical neglect were identified as potential risk factors for young BPD (16, 21, 30, 41, 42, 44, 45, 48). Particular attention was paid to chronic exposure to peer victimization (49–54, 60). Some authors highlighted the importance of gene-environment interaction in development of BPD. In fact, subjects with particular genotypes have a greater risk to develop BPD in presence of predisposing environment conditions (48).

With regard to child and adolescent-related factors, a number of studies identified as main predictors of BPD at an early stage the following temperamental traits: aggressiveness (in particular relational aggression) (55, 57, 61, 62), impulsivity, affective instability, negative affectivity (5, 19, 22, 45, 63–65), and low emotional control by interaction with maltreatments (21, 43, 68).

Several psychopathological conditions in childhood and adolescence that potentially predict BPD were examined. Results showed that both internalizing (depression, anxiety, dissociation, self-harming) and externalizing (substance use disorder, ADHD, opposite defiant disorder, conduct disorder) disorders are involved in promoting BPD onset in young people (25, 44, 45, 74–81).

Extensive overlap with internalizing and externalizing psychopathology in adolescence and early adulthood can produce noticeable difficulties in the diagnosis of BPD. The new alternative model of personality disorders proposed by *DSM-5* could contribute to address these difficulties as it combines the traditional categorical approach with a dimensional traits model that is likely more sensitive to specific traits of early onset BPD. Of course this is only a hypothesis that needs to be confirmed by data.

Findings from neuroimaging studies allow us to verify that in adolescents with BPD are already present some abnormalities that we can find in adulthood. Available studies investigated only the structural aspects, as functional brain imaging studies have not been conducted in adolescents to our knowledge. The most important abnormalities concern fronto-limbic structures. In particular, the reduction of volume of OFC (84, 86), ACC (89, 90), and hippocampal asymmetry (43) were found in early BPD compared with controls. Also in white matter, some specific alterations were observed: inferior longitudinal fasciculus and the fornix showed a diminished fractional anisotropy in BPD adolescents compared with controls. These findings suggested that abnormalities in specific white matter pathways involved in emotion regulation could indicate that a wider network of emotion processing is dysfunctional in adolescents with BPD (2).

Evidence collected on the impact of early BPD onset on later functioning of patients are generally in accordance to retain that BPD in childhood and adolescence predict a severe impairment of interpersonal and occupational functioning (99, 100), as well as younger age at first treatment, affective instability, antisocial behaviors, substance abuse, and dysfunctional relationships with parents (96–98). Furthermore, poor functional outcome persists up to 20 years into the future in individuals who presented BPD in adolescence (101). Some precocious protective factors related to childhood competence, such as higher IQ, good childhood work history, higher agreeableness, and lower neuroticism, were also identified (98, 103).

In conclusion, specific BPD features emerge in childhood and adolescence. Recognizing these precocious predictors may have significant clinical implications. Early onset of this complex and serious personality disorder is associated with high risk of negative outcome and long-term poor psychosocial functioning. Precocious identification of BPD symptoms and accurate investigation of protective and risk factors is fundamental to promote prompt and adequate intervention programs and to improve the natural life-course trajectory of the disorder.

### A Preliminary Model of Risk Factors in BPD

We tried to support the work of clinicians in this field by providing a synthetic summary of findings collected in the different clusters of risk factors. So, it should be easier to identify more common and significant associations in the clusters of environmental precocious factors, child and adolescent temperament and personality factors, early psychopathological features, and neuroimaging factors.

A further step that can be useful for clinicians to detect early clinical conditions and to implement preventive interventions consists in the proposal of a hypothetic model that represents a high-risk condition for the onset of BPD. This model is a combination of more important and common factors identified in literature and is supported by the idea that their interactive effects are stronger and more relevant than the separate effects of single factors. A reasonable hypothesis on the basis of available data is that high-risk subjects are characterized by a series of predisposing factors. The first factor to consider is a positive history of early traumatic experiences. According to the more common findings in literature, early trauma can be represented by conditions of abuse or neglect in childhood or adolescence, or can be the consequence of persistent abnormalities in familial behaviors and relationships due to severe mother psychopathology. The effects of traumatic experiences are substantially increased when they do not occur as isolated events, but when the dysfunctional familial environment that produces traumas interacts with the child’s innate temperamental features. In this case, authors have identified a significant role for three temperamental traits: impulsive aggression, inadequate emotional control, and negative affectivity. Another relevant factor that can combine its effects with the previously reported environmental and temperamental dysfunctions to enhance the risk of early onset BPD is the occurrence in childhood/adolescence of precocious internalizing and externalizing psychiatric disorders. Particular attention has been received by depression, ADHD, and substance use disorder, that all represent psychopathological conditions with a frequent onset in early age, but a long-lasting association with symptoms of BPD in adulthood. We can suggest that some of these disorders are not independent comorbidities, but must be conceptualized as precocious expressions of BPD pathology. A few studies indicated that studies of neuroimaging can contribute to identify which brain structures are altered in subjects with risk factors for early onset BPD. For example, structural abnormalities of fronto-limbic areas have been related to impulsive and emotional dysregulation. If these changes of brain structures are specific enough, they will contribute to identify biological markers or neural signatures, a primary goal in psychiatric and brain imaging research. Of course, it must be noticed that we present here only a hypothetical model with the main purpose to stimulate the interest of researchers and the debate among experts. The indicators of a high-risk condition for early onset of BPD, and particularly the effects of their coexistence and interaction in the proposed model, must be furtherly investigated and confirmed in specific studies. One of the more challenging issues at the present state of our knowledge is to make clear which of the factors proposed in this model have a primary role in the pathogenesis of BPD and which intervene only at a later time to augment and trigger the effects of primary factors.

An important contribution to understand the complex effects of temperamental traits, traumatic experiences, and environmental dysfunctions on the neurobiology of young BPD patients could derive from studies of functional changes in brain areas during administration of specific stimuli (108). For example, studies of autobiographical memories in such populations could be of great value to investigate the effects of life events and traumatic experiences on the function of fronto-limbic brain structures involved in the construction of identity.

## Author Contributions

PB and SB equally contributed to summarizing the literature data and writing the review. MB collected literature data and organized the tables. PR contributed to writing and supervising the review.

## Funding

This study was supported by Ministero dell’Istruzione, dell’Università e della Ricerca-MIUR projects “Dipartimenti di Eccellenza 2018-2022” to the Department of Neuroscience “Rita Levi Montalcini.”

## Conflict of Interest

The authors declare that the research was conducted in the absence of any commercial or financial relationships that could be construed as a potential conflict of interest.
